# Identification of candidate genes and mutations in QTL regions for chicken growth using bioinformatic analysis of NGS and SNP-chip data

**DOI:** 10.3389/fgene.2013.00226

**Published:** 2013-11-05

**Authors:** Muhammad Ahsan, Xidan Li, Andreas E. Lundberg, Marcin Kierczak, Paul B. Siegel, Örjan Carlborg, Stefan Marklund

**Affiliations:** ^1^Division of Computational Genetics, Department of Clinical Sciences, Swedish University of Agricultural SciencesUppsala, Sweden; ^2^Department of Animal and Poultry Sciences, Virginia Polytechnic Institute and State UniversityBlacksburg, VA, USA

**Keywords:** candidate genes, growth, functional prediction, genetic divergence, QTL, SNP, resequencing

## Abstract

Mapping of chromosomal regions harboring genetic polymorphisms that regulate complex traits is usually followed by a search for the causative mutations underlying the observed effects. This is often a challenging task even after fine mapping, as millions of base pairs including many genes will typically need to be investigated. Thus to trace the causative mutation(s) there is a great need for efficient bioinformatic strategies. Here, we searched for genes and mutations regulating growth in the Virginia chicken lines – an experimental population comprising two lines that have been divergently selected for body weight at 56 days for more than 50 generations. Several quantitative trait loci (QTL) have been mapped in an F2 intercross between the lines, and the regions have subsequently been replicated and fine mapped using an Advanced Intercross Line. We have further analyzed the QTL regions where the largest genetic divergence between the High-Weight selected (HWS) and Low-Weight selected (LWS) lines was observed. Such regions, covering about 37% of the actual QTL regions, were identified by comparing the allele frequencies of the HWS and LWS lines using both individual 60K SNP chip genotyping of birds and analysis of read proportions from genome resequencing of DNA pools. Based on a combination of criteria including significance of the QTL, allele frequency difference of identified mutations between the selected lines, gene information on relevance for growth, and the predicted functional effects of identified mutations we propose here a subset of candidate mutations of highest priority for further evaluation in functional studies. The candidate mutations were identified within the *GCG, IGFBP2, GRB14, CRIM1, FGF16, VEGFR-2, ALG11, EDN1, SNX6, *and* BIRC7 *genes*.* We believe that the proposed method of combining different types of genomic information increases the probability that the genes underlying the observed QTL effects are represented among the candidate mutations identified.

## INTRODUCTION

Economically important production traits in domestic animals are generally complex, i.e., determined by factors that may include both genetic and environmental regulators. This is also true for many diseases in humans and animals. Thus, while it is often highly desirable to understand the regulation of specific complex traits, the task can be extremely challenging. For example, regions identified by quantitative trait loci (QTL) analysis will even after fine mapping of the QTL typically indicate regions including millions of base pairs and hundreds of genes that need to be explored to find causative mutation(s).

In this study our aim was to develop a bioinformatics strategy to mine already identified QTL regions to identify candidate genes for growth trait in chicken. The QTLs have been identified for body weight at 56 days of age in the Virginia chicken lines – an experimental population comprising two lines that have been divergently selected for body weight at 56 days for more than 50 generations at Virginia Tech (Dunnington and Siegel, 1996; Marquez et al., 2010; Dunnington et al., 2013). Both lines started from the same base population, which was produced from crosses of seven partially inbred lines of White Plymouth Rocks and now differ by more than 10-fold in body weight at selection age. Individuals from the 41st generation of these High-Weight selected (HWS) and Low-Weight selected (LWS) lines were used as founders in a QTL mapping pedigree and several QTL regions were mapped in an F2 intercross between the lines (Jacobsson et al., 2005). These regions have subsequently been replicated and fine mapped using an Advanced Intercross Line (Besnier et al., 2011). Candidate genes and mutations were here sought in the regions of the QTLs where the greatest allele frequency differences between HWS and LWS founder lines of the QTL cross were observed by individual SNP-chip genotyping and next generation sequencing (NGS) of DNA pools from the HWS and LWS. Based on a bioinformatic analysis of these regions and the SNPs detected by NGS we present candidate genes and mutations of high priority for further investigations in order to explain the observed QTL effects.

## MATERIALS AND METHODS

**FIGURE 1:**
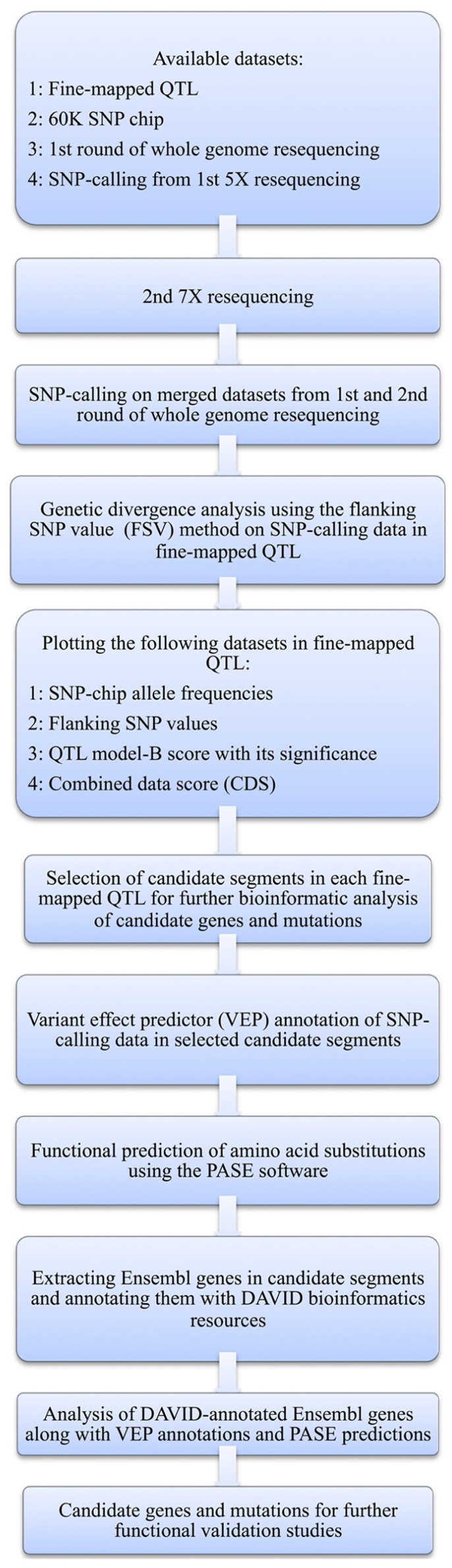
**Flow diagram of the bioinformatic analysis methods used here to identify candidate genes and mutations**.

Here, we present a bioinformatic strategy that in a structured and objective way helps to prioritize candidate genes for further study in mapped QTL regions by integrating information from multiple sources. First, the region to be evaluated further is narrowed down by, at each SNP-location in the evaluated region, calculating a combined score for the potential that each part of the region harbors a mutation underlying the phenotype. This is done by combining the statistical support from significance of the QTL effect at the particular marker, which is a measurement of the effect of the alternative alleles on the studied phenotype, with two measures of the genetic divergence between the founder lines (i.e., allele-frequency differences) at the particular location, which is an indicator of the direct or indirect selective pressure on the region due to an association with the phenotypes of importance when generating the divergent founder lines. Then, all the polymorphisms in the prioritized region are evaluated in more detail to select the most likely genes affecting the analyzed trait and bioinformatically predict the potential functional effects of each identified polymorphism. The details of the procedure, and its application to our particular chicken dataset, are described with a flowchart in **Figure 1** and in the text below.

### MAPPED QTL REGIONS TO BE EXPLORED

We studied seven fine-mapped QTL on chicken chromosomes 1–5, 7, and 20,with previously observed effects on body-weight at selection age in a QTL mapping pedigree founded with HWS and LWS chickens from generation 41 (Jacobsson et al., 2005; Besnier et al., 2011). The fine-mapping of the QTL was previously reported by Besnier et al. (2011) where the effect of each SNP in the QTL regions was estimated using a Flexible Intercross Analysis model (Rönnegård, 2008). The statistical QTL support curve across the regions from the analysis based on this model (Model B in the original paper) was here used for identification and evaluation of candidate regions.

### INDIVIDUAL GENOME-WIDE 60 K SNP CHIP GENOTYPING

Genome-wide 60K SNP chip genotypes of 20 individuals from each of the HWS and LWS lines, generation 41 (Marklund and Carlborg, 2010) was available. We used these genotype data to estimate the allele-frequency differences between the lines across the QTL regions to be explored.

### GENOME RESEQUENCING OF POOLED POPULATION-SAMPLES AND SNP-CALLING

Genome resequencing was performed in two separate runs using DNA pools from the HWS and LWS lines. The data from the two experiments were combined to maximize the sensitivity in the SNP detection.

For earlier studies DNA from two pools of genomic DNA, one from each of the HWS and LWS lines, were used to generate resequencing data with 5× average depth coverage for each line. The reads were aligned to the Red Jungle Fowl’s (RJF) reference genome assembly (WUGSC 2.1/galGal3, May 2006; Marklund and Carlborg, 2010; Rubin et al., 2010).

For the current and future studies the second round of resequencing was performed using two new pools of DNA samples. The individuals selected for each pool were guided by data from earlier performed 60K SNP-chip genome-wide genotyping. From each line, the eight individuals with the most non-representative genotype pattern in the QTL regions were selected to increase the possibilities for detection of variation within lines and thereby allow improved precision in the fine mapping of regions with high degree of between-line fixation. The ABI SOLiD resequencing was carried out by the Uppsala Genome Center using mate-pair libraries and 50 bases per read with ~7× depth coverage in each line. We aligned the reads to the RJF reference genome assembly (WUGSC 2.1/galGal3, May 2006) using the MOSAIK software (Lee et al., 2013) The resequencing datasets from the two rounds of sequencing were combined for SNP calling based on a total of ~12× depth coverage in each line. However in each line SNP alleles were called at each SNP position as determined using the threshold of three non-RJF reads that we set for SNP detection including the total number of reads from both lines (i.e., ~24× depth coverage) to increase the sensitivity. The GigaBayes software, a newer version of PolyBayes (Marth et al., 1999), was used for SNP calling.

### GENETIC DIVERGENCE ANALYSIS USING THE FLANKING-SNP-VALUE METHOD IN RESEQUENCING DATA

We applied the flanking-SNP-value (FSV) method (Marklund and Carlborg, 2010) to the resequencing data from the HWS and LWS lines across the selected QTL regions. The FSV method computes estimated allele frequency differences between the HWS and LWS lines for each evaluated SNP position based on information from the SNP itself as well as from data of flanking SNPs in both directions within an interval presumed to show a high degree of linkage disequilibrium with the SNP. Thus, the input data for FSV computation are the AB scores at all these positions, which in each line indicate the proportion of resequenced reads that are in agreement with reference sequence of RJF.

### A COMBINED SCORE FOR CANDIDATE GENE PRIORITIZATION

The allele frequency differences based on the individual SNP genotyping, the genetic divergence estimates (FSV) from the population-pool genome resequencing were plotted across the QTL regions together with the QTL support-curve from the QTL fine-mapping (Besnier et al., 2011). A combined data score (CDS) was also calculated based on these three information sources as:

$$\begin{array}{c} CDS=\{[(FSVscore+SNPchip-allele\,\;freq.)/2]\\ \;+(Normalized\,\;score\,\;of\,\;QTL_{-}ModelB)\}/2 \end{array}$$

The CDS was plotted to provide an objective statistic to prioritize regions for further analysis and evaluations of candidate genes and mutations. In most cases the regions were selected above the QTL significance and with high CDS.

### IDENTIFICATION OF CANDIDATE GENES AND MUTATIONS IN PRIORITIZED REGIONS

Genes were identified in the prioritized regions within the QTL using the Ensembl database (version 67; Flicek et al., 2012). The general functions and gene annotations for each gene was compiled using information from the Database for Annotation, Visualization and Integrated Discovery (DAVID; Huang et al., 2009a,b). DAVID integrates annotations for genes from different omics databases including, for instance, gene ontology (GO), KEGG and PANTHER.

All SNPs detected by resequencing in selected candidate regions were analyzed with variant effect predictor (VEP) from Ensembl (McLaren et al., 2010). VEP maps the locations of SNPs, insertions and deletions to different functional parts of Ensembl genes, transcripts and regulatory sequences. It differentiates coding SNPs in exons as synonymous or non-synonymous and shows amino acid substitutions. For some species, however not in chicken, it also predicts the functional consequences of non-synonymous SNPs (nsSNPs) on carrying proteins. We analyzed nsSNPs in protein coding sequences in the prioritized QTL regions using an in-house developed tool for prediction of amino acid substitutions based on their physicochemical properties (PASE) and evolutionary conservation (Li et al., 2013).

The DAVID annotated gene list was then filtered to identify the most likely candidate genes for growth in each QTL region. This was done by selecting the genes that had been associated with any of the following growth-related keywords: growth, development, morphogenesis, formation, proliferation, differentiation, regeneration, mineralization, elongation, biosynthetic, biogenesis, and organization. This set of terms was selected arbitrarily from ontology literature. The whole annotated gene list description was also reviewed to ensure no obvious candidates for growth were omitted.

## RESULTS

**FIGURE 2:**
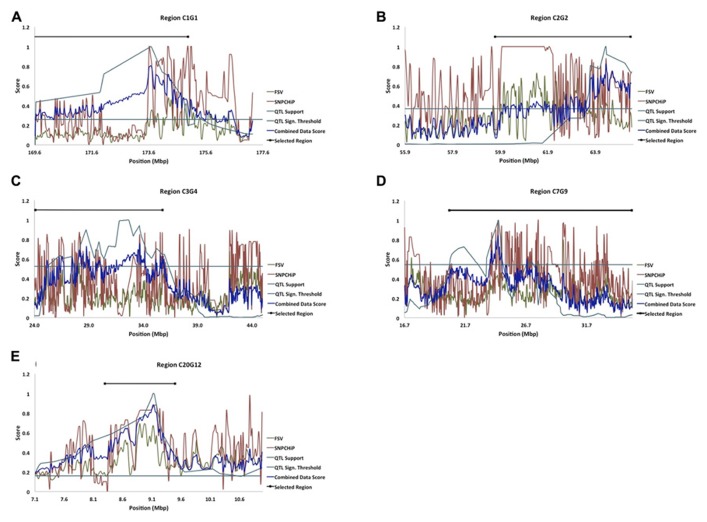
**(A–E)** Five of the fine-mapped growth QTL regions based on model B (QTL Support curve), and their significance threshold (QTL Sign. Threshold line) as in Besnier et al. (2011). The FSV curve represents FSV computations from resequenced NGS data from the HWS and LWS lines (Marklund and Carlborg, 2010), the SNP chip curve represents allele frequency differences between HWS and LWS from SNP genotyping, and the combined data score curve represents the formulated score from all of the above stated dataset curves. The Selected Region line represents the selected candidate regions for bioinformatic analysis of genes and mutations.

In an earlier study, Besnier et al. (2011) fine-mapped a number of QTL affecting body-weight at 8 weeks of age (**Table 1**; **Figures 2A–E**). The evaluated QTL regions are located on chicken chromosomes 1–5, 7, and 20 and cover in total 121.4 Mbp of the genome.

**Table 1:** Fine-mapped growth QTL regions with significance according to Besnier et al. (2011).

GGA^1^	QTL^2^	Region name	Start (Mbp^3^)	End (Mbp^3^)	Size (Mbp)
1	Growth1	C1G1	169.6	181.0	11.4
2	Growth2	C2G2	47.9	65.4	17.5
3	Growth4	C3G4	24.0	68.0	43.9
4	Growth6	C4G6	1.3	13.5	12.1
5	Growth8	C5G8	33.6	39.0	5.3
7	Growth9	C7G9	10.9	35.4	24.5
20	Growth12	C20G12	7.1	13.8	6.7
Total					121.4

1 GGA: *Gallus Gallus* Autosome;

2 QTL names as in Besnier et al. (2011);

3 Coordinates based on the Chicken (*Gallus gallus*) assembly v 2.1/galGal3

Using the prioritizations strategy described above, 44.7 Mbp of these original QTL were selected using the combined information from the QTL analysis and estimates of differences in allele frequencies between the lines inferred from SNP chip genotyping and FSV computation (**Table 2**).

**Table 2:** Candidate regions selected based on QTL data and allele frequency differences between the lines inferred from SNP chip genotyping and FSV computation from resequencing. The selected percentages of the QTL regions significant with model B, are given (Besnier et al., 2011).

Region name	Start Mbp^1^	End Mbp	Size (Mbp)	QTL support^2^	Ensembl genes^3^
C1G1	169.6	175.0	5.4	5.4	97
C2G2	59.7	65.4	5.7	2.1	52
C3G4	24.1	35.8	11.7	10.3	142
C4G6	10.6	12.9	2.3	0.0	62
C5G8	34.2	36.8.	2.6	0.0	20
C5G8	38.2	39.0	0.8	0.0	16
C7G9	20.4	35.4	15.0	4.3	209
C20G12	8.3	9.5	1.2	1.2	38
Total			44.7	23.3	636

1 Coordinates based on the Chicken (*Gallus gallus*) assembly v 2.1/galGal3;

2 Size of the selected regions significant with QTL model B (Besnier et al., 2011);

3 Number of Ensembl genes in the initial list in the selected regions

In **Table 3**, we provide a summary of the results obtained using the Ensembl VEP tool. Nearly 61,000 SNPs (excluding intergenic and intronic SNPs) were found to be located within functional elements across the selected candidate segments in this analysis. In **Table 4**, we provide a selection of one or two of the best candidate mutations in each region.

**Table 3:** The variant effect predictor summary of SNPs in selected candidate segments of the QTL regions (according to **Table 2**).

Location within gene	Region							
	C1G1	C2G2	C3G4	C4G6	C5G8	C7G9	C20G12	Total
3Prime UTR	200	93	200	153	73	348	75	1142
3Prime UTR, Splice site		1			1			2
5Prime UTR	22	9	44	20	3	50	28	176
5Prime UTR, Splice site				1		4		5
Coding unknown			1			1		2
Downstream	6118	2636	5318	2373	1395	7930	1384	27154
Essential splice site	2	3	6	1	1	4	3	20
Non-synonymous coding	215	82	255	92	60	470	80	1254
Non-synonymous coding, Splice site	6	4	8	5	3	17	1	44
splice site, Intronic	78	37	133	33	24	191	31	527
Stop gained	5		7	3	2	10		27
Stop gained, Non-synonymous coding	1							1
Synonymous coding	350	208	543	165	99	1113	159	2637
Synonymous coding, splice site	9	9	12	5	6	20	12	73
Upstream	5506	2626	5755	2570	1200	8312	1479	27488
Within mature miRNA						1		1
Within non-coding gene	4		2	1	3	12	4	26
Within non-coding gene, splice site						1		1
Total	12516	5708	12284	5422	2870	18484	3256	60540

**Table 4:** Candidate mutations identified in the evaluated QTL regions.

Region	SNP (bp)^1^	Gene	SNP location^2^	No of AA^3^ reads; depth coverage	Qual^4^	AFD^5^	PC Score^6^	EC Score^7^	PE Score^8^
C1G1	174634021	Asparagine-linked glycosylation 11 homolog (*ALG11*)	CpG island, upstream	7; 10	72	0.97	N/A	N/A	N/A
C2G2	63823523	Endothelin 1(*EDN1*)	CpG island, upstream	3; 13	53	0.95	N/A	N/A	N/A
C3G4	33678270	Cysteine rich transmembrane BMP regulator 1 (*CRIM1*)	Protein code, NS K/I	10; 19	182	0.97	0.67	0.63	0.42
C4G6	12044024	Similar to receptor tyrosine kinase (*VEGFR-2*)	CpG island, upstream	4; 8	82	0.97	N/A	N/A	N/A
C4G6	12902414	Fibroblast growth factor 16 (*FGF16*)	CpG island, downstream	8; 16	175	0.95	N/A	N/A	N/A
C5G8	38316301	Sorting nexin 6 (*SNX6*)	CpG island, upstream	8; 14	142	0.97	N/A	N/A	N/A
C7G9	21686625	Growth factor receptor-bound protein 14 (*GRB14*)	CpG island, downstream	3; 12	52	0.97	N/A	N/A	N/A
C7G9	22711910	Glucagon (*GCG*)	CpG island, downstream	3; 9	46	0.87	N/A	N/A	N/A
C7G9	24802616	Insulin-like growth factor binding protein 2 (*IGFBP2*)	Protein code, synonymous, CpG island	4; 8	69	0.95	N/A	N/A	N/A
C20G12	8715398	Baculoviral IAP repeat-containing 7 (*BIRC7*)	Protein code, NS I/V	5; 8	65	0.97	0.29	0.14	0.04

1 Coordinates based on the Chicken (*Gallus gallus*) assembly v 2.1/galGal3;

2 Location of the SNP in gene and also amino acid substitution in case of non-synonymous (NS) SNP;

3 Total number of reads in both lines representing the alternate allele (AA) versus the total depth coverage across the SNP position;

4 The Phred scaled probability that a REF/ALT polymorphism exists at this site given sequencing data. Because the Phred scale is -10 * log(1 - *p*), a value of 10 indicates a 1 in 10 chance of error, while a 100 indicates a 1 in 10^10^ chance;

5 Allele frequency difference between the chicken lines as estimated using the GigaBayes software given that a total of 19 individuals from each line were included in the pools;

6 Physico-chemical score of amino acid substitution calculated using PASE (Li et al., 2013).

7 Evolutionary conservation score of amino acid substitution calculated using PASE (Li et al., 2013);

8 Combined score of PC and EC of amino acid substitution calculated using PASE (Li et al., 2013).

## DISCUSSION

In this study we have developed and applied a bioinformatic strategy to search for candidate mutations affecting body weight at 56 days in several QTL regions that were previously identified and fine-mapped in an intercross between two divergently selected chicken lines. Given the 40 generations of divergent selection for body weight it is reasonable to assume that many of the underlying functional mutations will display a relatively large allele frequency difference, or complete fixation, between the lines. This assumption is supported by earlier work with the lines that many regions across the genome have been driven to fixation for alternative alleles in the lines and that most selection has been on standing genetic variation present in the common base-population at the onset of selection (Johansson et al., 2010). At a smaller number of selected loci mutations might have arisen after the initiation of selection. It is, however, unlikely that the QTL evaluated here are due to such new mutations as they are identified using a statistical analysis that assumes that the crossed lines are fixed for alternative QTL alleles.

To narrow down the target regions and identify the most plausible mutations, we used several independent sources of information. First, measurements of the genetic divergence between the founder lines of the intercross were used as indicators of regions that have been under strongest selection. Both individual SNP chip genotyping and genome resequencing of pools of individuals were used to provide stability and high-resolution in the estimates of the allele frequency difference between the lines.

The potential functional impact of genes and SNPs located within the target regions was bioinformatically evaluated to identify a set of candidate mutations to be further tested and evaluated in functional studies. In regions where there exist several possible candidate genes, our use of a combined and objective selection criteria helped to localize the most promising candidate genes and mutations. The genes and mutations listed in **Table 4** qualified as the strongest candidates underlying the observed QTL. Among these, the glucagon (*GCG*) gene on chromosome 7 (C7G9 region) is perhaps the most obvious candidate gene due to its well-documented effect on appetite (Suzuki et al., 2010), a trait for which the HWS and LWS lines show an extreme difference. No non-synonymous mutations were found in the glucagon gene, but a mutation was identified in a downstream CpG island with a large (0.87) estimated allele frequency difference between the lines (AFD), and possibly a regulatory effect on glucagon gene expression. The C7G9 region also included mutations in CpG islands with even larger AFD estimates and possibly regulatory roles in genes that in turn can regulate other genes with effects on body weight. Such mutations were found in the insulin-like growth factor binding protein 2 (*IGFBP2*) and the growth factor receptor-bound protein 14 (*GRB14*; e.g., Holt and Siddle, 2005) genes. The *IGFBP5* gene is also located in this target region but at this stage we have not found sufficient support for any strong candidate mutation in that gene. The IGF binding proteins can specify the actions of insulin-like growth factors which have key roles in vertebrate growth and development (e.g., Wood et al., 2005). Interestingly, the possibly regulatory *IGFBP2* mutation reported here is located in a coding sequence that is a part of a CpG island. Even though it is a synonymous mutation it may affect the *IGFBP2* expression through mechanisms of codon usage, GC content and/or mRNA stability and folding (reviewed by Shabalina et al., 2013). Overexpression of *IGFBP2* has been shown to reduce postnatal body weight gain in transgenic mice (Hoeflich et al., 1999). The *GRB14* gene encodes a cellular adapter protein that can bind to receptor tyrosine kinases and intracellular proteins and thereby be involved in various processes. For example, it can bind and modify the signals from the insulin receptor and insulin-like growth factor 1 and its implication in growth regulation has been shown (reviewed by Holt and Siddle, 2005).

Strong candidate genes and mutations were also found in QTL regions on chromosome 3 (C3G4) and 4 (C4G6). In the C3G4 region, the gene encoding the cysteine rich transmembrane BMP regulator 1 (*CRIM1*),showed a non-synonymous mutations with large allele frequency difference between the lines and high PE scores (i.e., combined PC and EC scores; **Table 4**) with the PASE tool. CRIM1 interactions with growth factors may be important for the development of the central nervous system (CNS) and other organs (Kolle et al., 2000). Perhaps most interesting is the impact the *CRIM1* gene possibly has on the CNS because Ka et al. (2009) reported genes that regulate neuronal plasticity to be differentially expressed between the HWS and LWS lines in the brainstem and hypothalamus. Moreover, electrolytic hypothalamus lesions has been shown to increase appetite in the LWS but not in the HWS line which further supports that CNS is highly involved in the differences between these chicken lines (Burkhart et al., 1983).

In the C4G6 region, candidate CpG island mutations were identified within the fibroblast growth factor 16 (*FGF16*) and vascular endothelial growth factor receptor 2 (*VEGFR-2*) genes. *FGF16* is known to be involved in embryonic development and cell growth (Antoine et al., 2006) whereas the *VEGFR-2 *gene has been reported to be of importance for angiogenesis (Patterson et al., 1995).

In the chromosome 1 QTL region (C1G1) we also found a candidate mutation, possibly regulatory, in the asparagine-linked glycosylation 11 homolog (*ALG11*) gene. *ALG11* has been reported to be involved in biosynthetic processes and required for normal growth in yeast (Cipollo et al., 2001).

The chromosome 2 QTL region (C2G2) showed CpG island mutations at the endothelin 1 (*EDN1*) gene with the two chicken lines fixed for opposite alleles. EDN1 is known for roles in regulation of blood pressure and development (Kurihara et al., 1994).

In the regions on chromosome 5 (C5G8) and 20 (C20G12) the genes found in the analysis were less obvious candidates. However, such genes may still have key roles in processes with complex and indirect effects on growth-related traits. Keeping this in mind, we consider mutations identified in the sorting nexin 6 (*SNX6*; Caldwell et al., 2005; C5G8 region) and baculoviral IAP repeat-containing 7 (*BIRC7*; Kasof and Gomes, 2001; C20G12 region) genes are of most interest to investigate further.

In conclusion, the described combination of data from QTL mapping, next-generation sequencing, SNP chip genotyping and bioinformatic analysis has provided a list of plausible candidate genes and mutations that will facilitate further verification and experimental evaluation. The support for this list from different types of data and analysis enhances the probability that the selected genes and mutations underlying the QTL effects are an unbiased selection of genes and that the contributing gene(s) are included in the set. Further studies based on this list may therefore reveal mutations which underlie the observed QTL effects and can increase our understanding of growth regulation as well as be more emphasized in animal breeding programs with genomic selection.

## AUTHORS CONTRIBUTIONS

Muhammad Ahsan and Xidan Li carried out the region-targeted computation and analysis using the different sources of data and took part in the planning of the study. Marcin Kierczak and Andreas E. Lundberg performed the assembly of the SOLID resequencing datasets. Stefan Marklund initiated and planned the study. Paul B. Siegel and Örjan Carlborg contributed with comments and advice. Muhammad Ahsan and Stefan Marklund drafted the manuscript and all co-authors contributed to the final version.

## Conflict of Interest Statement

The authors declare that the research was conducted in the absence of any commercial or financial relationships that could be construed as a potential conflict of interest.
